# Editorial: Insights in social psychiatry and psychiatric rehabilitation: 2021

**DOI:** 10.3389/fpsyt.2022.1023972

**Published:** 2022-09-06

**Authors:** Antonio Vita

**Affiliations:** School of Medicine, Spedali Civili Hospital, University of Brescia, Brescia, Italy

**Keywords:** social psychiatry, psychiatric rehabilitation, physical activity, innovative methods, psychotherapy, artificial intelligence

The journey to psychiatric rehabilitation, once prerogative of few brave explorers in the field of social psychiatry, is now being open to new different and ambitious trajectories, where new paths such as physical activity and artificial intelligence add to more consolidated approaches such as Psychodynamic and Cognitive Behavior Therapy, through collaborative research, attention to stigma and personal perspective of young mental health professionals.

This Research Topic presents some interesting insights about different routes, that may represent ambitious challenges for the future of rehabilitation. We start with a review by a Canadian group (Ziebart et al.) that investigated how exercise and physical activity could influence psychotic symptoms, in people with psychosis, within a hospital setting. Twenty-four trials were included in the systematic review and 9 of them were included in the meta-analysis (1,426 participants). The results showed that aerobic exercise, but not yoga, was associated with reduction of psychosis symptom severity scores, when compared to usual care.

Then, shifting to artificial intelligence, Mouchabac et al. published a paper in which they evaluate the possible implementation of artificial intelligence (AI) within Clinical Decision Support System (CDSS) and how it may help in the complex decisions making process that is often needed in situations covered by Psychiatric Advance Directives (PADs). After analyzing the use of new information technology tools and techniques for the improvement of the patient's hospital experience, also focusing on ethical issues, the authors conclude that AI should remain a decision support system as a partner of each party of the PAD contract. Patient's informed choice on the possibility to benefit of AI is central.

Talking about psychotherapy, a German team (Muschalla et al.) compared patients receiving Psychodynamic therapy (PDT) and Cognitive behavior therapy (CBT). The study involved 73 cognitive behavior therapists and 58 psychodynamic psychotherapists, who reported about 188 CBT patients and 134 PDT patients. No significant socio-demographics differences emerged between PDT and CBT patients. Number of sessions was lower in CBT patients, who also showed longer duration of illness, more parallel medical treatments and higher rates of sick leave. Correspondingly, CBT therapists reported more sociomedical interventions, like interdisciplinary treatment, work oriented interventions, and social support. The authors conclude that the differences between PDT and CBT may be explained by the fact that PDT requires analytical capabilities on the side of the patient, which may exclude patients with social problems, with complex mental disorders like dementia, or with a lack of psychological insight. The authors consider CBT as a more problem-oriented intervention, that allows to treat a wider range of patients.

Another interesting perspective has been proposed by Beeker et al., that published a paper providing an in-depth description about a 3-years collaborative project that took place in the context of a mixed-method process evaluation of innovative models of psychiatric care in Germany. Researchers interviewed each other about specific topics and personal experiences, thus collecting and re-discussing the results. This method, called “interactive interviewing” allowed researchers to reflect on their collaboration at different levels. According to the authors an atmosphere of mutual trust and respect within the group is crucial, and continuous self-reflection or supervision can be largely beneficial, while emerging conflicts should be considered as opportunities for personal growth and transformation.

The attention to mental health professionals and to their personal perspective in the field of rehabilitation is also demonstrated in other papers published in this Research Topic. Koelkebeck et al. reflected upon the research opportunities of psychiatric trainees and early career psychiatrists (ECPs). Two hundred and fifty-eight European early career psychiatrists, from 34 countries responded to a questionnaire regarding their research experience. Even if most participants were highly interested in research and highly satisfied with mentoring and publishing papers, they reported major obstacles toward their research activities, such as lack of time and funding.

In this context, the issue of stigma was investigated from the perspective of young nurses: a Chinese research team (Wang et al.) validated a translated version of a 20-item scale for assessing stigma of mental illness in nursing (SASMIN). The scale includes three dimensions: Violence/Dangerousness, Disability, and Irresponsibility/Lack of Competence. Five hundred and one nursing students participated in the study. The scale allowed to identify the different dimensions of stigma associated with mental illness among nursing staff and thus may be used in the clinical practice to improve quality of care and patient's satisfaction.

Finally, the issue of recovery from homelessness was analyzed by a Canadian study (Durbin et al.) focused on recovery education for people transitioning from homelessness, in the context of services provided by Recovery Education Centers (RECs). The authors compared recovery outcomes of adults with history of homelessness and mental health challenges enrolled in a REC, to those of a matched control group receiving usual interventions. Mean change in perceived empowerment at 1 year from baseline was not significantly different between groups. However, in a *post-hoc* analyses comparing subgroups with 1–13 h and 14+ h of REC participation the group receiving 14+ h of intervention showed greater change in perceived empowerment compared to controls. Mean change in mastery was also significantly different for the intervention subgroup with 14+ h of REC compared to controls. In conclusion when applied with enough intensity, recovery education may be a helpful add-on to health and social services for homeless people with mental health problems.

From the rocky mountains of stigma, to the open fields of physical exercise and psychotherapies, to the deep space of artificial intelligence, passing through the long and impervious road of recovery, and early career research challenges, this Research Topic is a path that is worth walking (and reading).

## Author contributions

The author confirms being the sole contributor of this work and has approved it for publication.

## Conflict of interest

The authors declare that the research was conducted in the absence of any commercial or financial relationships that could be construed as a potential conflict of interest.

## Publisher's note

All claims expressed in this article are solely those of the authors and do not necessarily represent those of their affiliated organizations, or those of the publisher, the editors and the reviewers. Any product that may be evaluated in this article, or claim that may be made by its manufacturer, is not guaranteed or endorsed by the publisher.

